# Neurofilaments Light Chain in Neurodegenerative Dementias: A Review of Imaging Correlates

**DOI:** 10.3390/brainsci14030272

**Published:** 2024-03-13

**Authors:** Chiara Gallingani, Chiara Carbone, Manuela Tondelli, Giovanna Zamboni

**Affiliations:** 1 Department of Biomedical, Metabolic and Neural Sciences, University of Modena and Reggio Emilia, 41125 Modena, Italy; 2 Neurology Unit, Azienda Ospedaliero Universitaria di Modena, 41126 Modena, Italy

**Keywords:** neurofilaments light chain, MRI, dementia

## Abstract

Neurofilaments light chain (NfLs) are currently recognized as a marker of axonal injury and degeneration. Their measurement in biological fluids has a promising role in the diagnosis, prognosis, and monitoring of the therapeutic response in neurological diseases, including neurodegenerative dementias. In recent years, their relationship with clinical phenotypes and measures of disease severity has been extensively studied. Here, we reviewed studies investigating the association between NfLs and imaging measures of grey matter (GM) and white matter (WM) damage in neurodegenerative dementias. We identified a large number of studies investigating this association in Alzheimer’s disease (AD) and disorders of the frontotemporal dementia (FTD) spectrum. Results were heterogeneous, possibly due to different methodological approaches—both in NfL measurements and imaging analyses—and inclusion criteria. However, a positive association between NfL levels and GM atrophy, WM microstructural disruption, glucose hypometabolism, and protein accumulation emerged invariably, confirming the role of NfLs as a reliable biomarker for neurodegenerative dementias, albeit not specific.

## 1. Introduction

Neurofilaments (NFs) are neuronal-specific heteropolymers belonging to the class of intermediate filaments (diameter 10 nm), which are important components of the neuronal cytoskeleton. Three main NF isoforms can be distinguished based on molecular weight: neurofilaments light (NfLs), medium (NfMs), and heavy (NfHs) chain. In addition to these isoforms, a-internexin in the central nervous system (CNS) and peripherin in the peripheral nervous system (PNS) can be included in the NF structure. These five proteins co-assemble into NFs in different combinations and concentrations depending on the type of neuron, location in the axon, and stage of development [1]. NFs can be found principally in large myelinated axons, where they play a fundamental role in maintaining axon caliber, ensuring radial growth, and the transmission of electrical impulses. Normally, they are highly stable in axons and their turnover is low. When axonal damage or degeneration occurs, they are released in large quantities into the interstitial space, from where they first pass into the cerebrospinal fluid (CSF) and then enter the blood [2]. Among the different isoforms, NfLs are the most abundant and soluble and can therefore be detected in biofluids [3]. Their levels increase irrespective of the specific neuropathological damage that has determined axonal loss. For these reasons, they are now recognized as a non-specific marker for axonal damage and neurodegeneration and represent a promising biomarker in neurological conditions [2,3].

In recent years, the methodological approaches to NfL detection in biofluids have evolved significantly. The enzyme-linked immunosorbent assay (ELISA) and the more sensitive electrochemiluminescence (ECL) assay are reliable technologies for measuring NfLs in CSF but they are not able to detect NfLs in blood, where their concentration is 40 times lower [3]. The first technology to enable accurate measurement of blood NfLs was the single-molecule array (Simoa) system, which utilizes microwells and paramagnetic microbeads to capture single antibody–antigen complexes. Thanks to this procedure, fewer NfL molecules are able to produce a detectable signal [4]. The Simoa assay showed 126- and 25-fold higher sensitivity than the ELISA and ECL assays, respectively [5], and demonstrated reproducible preanalytical and analytical performances, so it is now considered a reference method. However, standardized procedures and reliable clinical thresholds are still lacking, limiting their implementation in clinical practice.

A moderately strong correlation between CSF and blood NfLs has been demonstrated, suggesting that the less invasive detection of NfLs in blood can be considered a good indicator of the neuroaxonal damage happening in the CNS [6]. However, several confounding factors that affect NfL levels in blood have been identified. These include cardiovascular risk factors, body mass index, pregnancy, unrecognized head traumas, and renal function [4]. Among all, age has been identified as one of the most relevant influencing factors, to the extent that age-specific reference values have been proposed [7]. Higher age is associated with higher NfL levels and higher variability among individuals. Studies have shown that CSF NfL levels increase 2.5-fold between the ages of 20 and 50 and successively double by the age of 70. Blood NfL levels have also been shown to increase by 2.2% per year between the ages of 18 and 70 [8]. Such age-related increases could be driven by different mechanisms, including the presence of more co-morbidities, higher permeability of the blood–brain barrier, and increased neuronal apoptosis in the elderly [4,8].

In the past few years, the role of NfLs as fluid biomarkers has been extensively studied in a variety of neurological conditions, from multiple sclerosis to head trauma. However, it is in the field of neurodegenerative diseases such as Alzheimer’s disease and frontotemporal dementia, where blood biomarkers are still lacking, that they have acquired particular relevance.

In Alzheimer’s disease (AD), both CSF and plasma NfLs are increased. This has been shown in patients with cognitive impairment that is severe enough to interfere with everyday independence (i.e., dementia due to AD), as well as, to a lesser extent, in milder phases of cognitive decline (i.e., mild cognitive impairment) due to AD [9]. Studies have demonstrated that the accuracy of plasma NfLs in distinguishing AD dementia from healthy controls is close to the accuracy of established CSF AD biomarkers (total tau, phosphorylated tau, and β-amyloid (Aβ)) and higher than the accuracy of plasma tau [9]. However, differentiating between AD (either in the MCI or dementia phase) and other neurodegenerative dementias is more challenging, revealing the low specificity of NfLs. Thus, it has been proposed that, for diagnostic purposes, blood NfLs should have a role as a screening tool to detect individuals at higher risk of having AD pathology who then need to undergo testing of more specific diagnostic biomarkers [10]. NfL elevation in CSF has also been associated with a greater risk of developing MCI in cognitively unimpaired individuals, with faster rates of cognitive decline in MCI, and with overall decreased survival in AD dementia, suggesting their role as a prognostic biomarker [11]. In familial AD, NfLs have been shown to be higher in mutation carriers than in non-carriers: their annual rate of change increases in mutation carriers as early as 16 years before the estimated symptom onset [12]. In both familial and sporadic AD, the rate of change increases closest to symptom onset. Longitudinal measurements of NfLs could therefore have a role as a biomarker of phenoconversion. Finally, their measurement has been introduced in several pharmacological trials on disease-modifying treatments for AD, showing their role as a biomarker of response to therapy [3].

The search for new biomarkers is even more important in the frontotemporal dementia-amyotrophic lateral sclerosis (FTD–ALS) spectrum, where specific fluid biomarkers are lacking, and imaging biomarkers are often insufficient for diagnostic and prognostic purposes. Among all neurodegenerative diseases, ALS has been shown to have the greatest elevation of CSF NfLs, since ALS patients present up to seven-fold higher levels than controls. This has been attributed to the massive degeneration of motor neurons, which have largely myelinated axons containing a great amount of NfLs [13]. In ALS, CSF and serum NfL levels correlate moderately with the disease progression rate and are not associated with the spatial distribution of the disease. For these reasons, they have been proposed as a biomarker to distinguish ALS from ALS-mimics and to discriminate between patients with rapid or slow progression ALS [13]. As for FTD, the first reports of NfL elevations in the CSF of patients with FTD date back to 1999 [14,15]. Since then, several studies have reported higher NfL concentrations in FTD patients compared to controls, in both the behavioral and language phenotypes (i.e., primary progressive aphasia (PPA)), as well as in FTD–ALS individuals [16,17,18,19]. Some data suggest a more prominent elevation in the semantic variant primary progressive aphasia (svPPA). It has been proposed that this difference reflects the association between svPPA and the TDP-43 pathology, which has shown higher NfL levels than the tau pathology [17,18]. The role of NfLs has also been investigated in familial FTD, where they are increased in each genetic group (i.e., C9ORF72 repeat expansion, MAPT, and GRN mutations). Symptomatic patients present higher NfL levels compared to both healthy controls and pre-symptomatic carriers. Higher baseline NfL levels in pre-symptomatic carriers than in non-carriers can be found from the age of 48, when some degree of axonal damage starts reflecting a prodromal stage of the disease. Moreover, similarly to familial AD and ALS, NfL levels present a higher increase rate near clinical onset [20], representing a useful tool for detecting converters who could benefit from a disease-modifying treatment. In fact, in both ALS and FTD clinical trials, NfLs are being used not only to monitor the response to treatment but also to identify pre-symptomatic patients in proximity of conversion who would be eligible for treatment. NfL levels correlate to greater disease severity, lower scores at cognitive tests, and shorter survival, suggesting that they may help differentiate FTD patients who will progress over time from those who have a clinical diagnosis of FTD but do not progress over time, indicated as “phenocopies” [3]. Initially, NfLs have been proposed as a marker not only to distinguish FTD patients from healthy controls but also to help in the differential diagnosis between FTD and AD or Lewy body dementia (LBD). However, recent studies have shown an overlap in NfL levels in these syndromes, therefore their diagnostic role has been reduced [21,22]. The same does not apply to the role of NfLs in the differential diagnosis between behavioral variant FTD (bvFTD) and primary psychiatric disorders (PPDs). In fact, bvFTD patients clearly present higher CSF and blood NfL levels than patients with PPDs, enabling discrimination between the two [23,24,25,26,27]. The relevance of these findings has been included in a consensus paper of the Neuropsychiatric International Consortium for Frontotemporal Dementia (NIC-FTD), where NfLs have been proposed as a diagnostic biomarker to distinguish between bvFTD and PPDs [28].

Less is known about the role of NfLs in other types of dementias. In LBD and atypical parkinsonism—progressive supranuclear palsy (PSP), corticobasal syndrome (CBS), and multiple system atrophy (MSA)—NfLs appear to be higher than in controls, predict disease progression, and allow differentiation from Parkinson’s disease (PD) [29,30].

In the past few years, the measurement of NfLs has gained increasing interest in the field of neurodegenerative diseases as a promising biomarker for diagnosis and prognosis, as well as for monitoring therapeutic responses. However, we still need a deeper understanding of this marker’s role in each of these situations before it can become part of the clinical routine. Studying the correlation between NfLs and neuroimaging features may help to better understand their disease-specific features and underlying mechanisms [13,31]. The present scoping review is aimed at collecting findings on this topic to sum up the present knowledge in this field, allowing for a more systematic understanding that will help clinical interpretation.

## 2. Methods

### 2.1. Search Strategy

For this scoping review, we performed a literature search of the MEDLINE/PubMed and Web of Science databases to identify eligible published articles from their inception to 9 November 2023. The following search terms were used: ((“neurofilament light”) OR (“neurofilaments light”) OR (“NfL”) OR (“NfLs”) OR (“neuro filament light”)) AND (((“brain volume”) OR (“brain density”) OR (“grey matter”) OR (“GM”) OR (“gray matter”) OR (“atrophy”) OR (“cortical thickness”)) OR ((“white matter”) OR (WM) OR (“microstructural integrity”) OR (“fractional anisotropy”) OR (FA) OR (“mean diffusivity”) OR (MD))).

Retrieved articles were imported into Rayyan, an online research tool for screening and data extraction in review studies. Two authors (CC and CG) independently screened titles and abstracts to identify eligible articles. Full texts of selected studies were then evaluated and non-eligible articles, as per established criteria, were excluded. Discrepancies in the selection were discussed with the senior author and neurologist (GZ) until a consensus was reached.

### 2.2. Inclusion and Exclusion Criteria

We included studies in English with available abstracts and full text, which evaluated the association between NfLs and neuroimaging features in the commonest neurodegenerative dementias, encompassing AD, FTD spectrum, and LBD. We directly searched for magnetic resonance imaging (MRI)-based studies, exploring structural and functional grey matter (GM) and white matter (WM) parameters; however, positron emission tomography (PET)-based studies that emerged from our search were considered eligible too.

Studies on the following diseases were excluded: multiple sclerosis, motor neuron diseases, α-synucleinopathies without dementia (i.e., PD and MSA), Huntington’s disease, traumatic brain injury, stroke, infections (including HIV and COVID-19), Down’s syndrome, alcohol assumption, and other non-neurodegenerative conditions (including VaD). We also excluded animal-based studies, case reports, and clinical trials, as well as non-original research (editorials and letters in response to previous articles) and abstracts or conference proceedings. Review articles were examined, but not directly included.

## 3. Results

### 3.1. Study Selection and Characteristics

**Figure 1:**
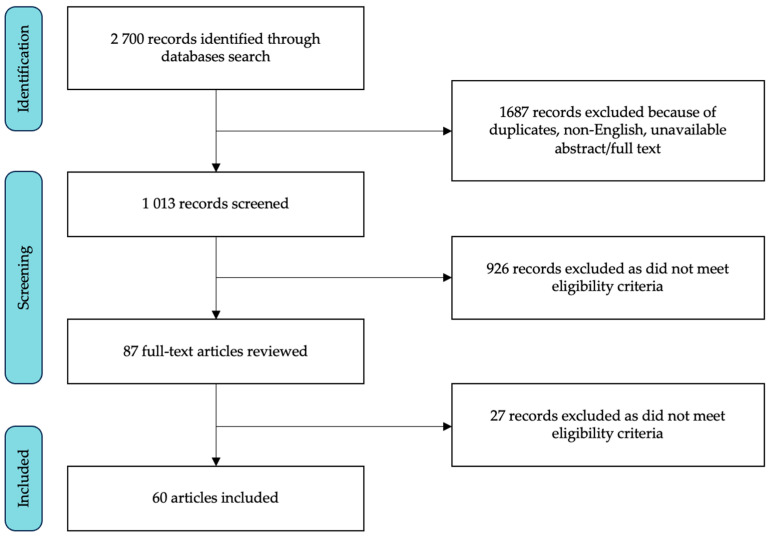
Flowchart of the study selection process.

The database search identified 2700 articles; we first excluded duplicates and studies published in non-English languages or with no available abstract/full text. The titles and abstracts of the remaining 1013 studies were reviewed and 926 articles were excluded since they were not related to the topic of interest or did not meet the inclusion criteria. The remaining 87 articles underwent full-text review, and 27 more articles were ruled out. Finally, 60 studies were deemed eligible. The flowchart of screened and selected studies is shown in Figure 1; the main details of included studies are reported in Table 1.

Twenty-four studies focused on AD, 19 on FTD spectrum, and 4 on both diseases. We also found 13 articles on MCI patients, independently from their underlying neuropathological processes. No studies on LBD patients were found. Thirty-one studies had a cross-sectional design, 2 had a longitudinal design, and 27 had both a cross-sectional and longitudinal design. Among all the studies, NfL levels were measured in the blood in 28, in the CSF in 20, and in both blood and CSF in 12.

### 3.2. Alzheimer’s Disease

Twenty-eight studies investigated the association between NfLs and different clinical measures of disease severity in AD, including imaging measures, both with cross-sectional and longitudinal designs. Among these studies, 11 used data from the Alzheimer’s disease neuroimaging initiative (ADNI) or similar convenience cohorts, including healthy controls, MCI patients, and AD patients classified based on clinical criteria. Only a subset of individuals in these studies had undergone CSF measurement of AD biomarkers, so that Aβ positive and negative subjects were frequently analyzed together.

Zetterberg et al. [54] reported a positive correlation between CSF NfLs and hippocampal atrophy, at baseline and over time, and a positive correlation with ventricular volume and whole-brain longitudinal atrophy in a single group encompassing AD patients, MCI patients, and controls from the ADNI database. Working on the same cohort, Mattsson et al. [9,42] found a correlation between higher plasma NfLs at baseline and both baseline and subsequent larger ventricular volumes, smaller hippocampal volumes, and thinner cortices in temporo-occipital areas (i.e., entorhinal, inferior temporal, middle temporal, and fusiform cortices). The same authors also confirmed these results on CSF samples, dividing subjects into Aβ+ and Aβ- (same results in the two groups) [42]. When dividing the three clinical groups, longitudinal plasma NfL increases were associated with ventricular volume expansion in AD, MCI, and control groups, and with accelerated loss of hippocampal volume and entorhinal cortical thickening in MCI patients and controls [43]. Benedet et al. [33] found that plasma NfLs negatively correlated with frontal and hippocampal GM and fronto-parietal WM volumes in healthy controls, and with wider fronto-temporal GM areas and whole-brain WM volumes in MCI and AD patients, at baseline and over time. Focusing on similar groups of subjects, Chen et al. [35] found that higher CSF NfLs were associated with hippocampal atrophy in MCI patients and controls, and also with ventricular enlargement in AD patients; also, plasma NfLs were associated with the same measures in all the groups.

Dhiman et al. [38] used data from the Australian imaging biomarkers and lifestyle study of ageing (AIBL) database, again dividing participants on the basis of clinical criteria, but performing lumbar puncture to collect CSF NfLs. They found that among controls, MCI, and AD patients CSF NfLs were negatively correlated with whole-brain and hippocampal GM volume. In a similar group and also in Aβ+ AD patients alone, Boerwinkle et al. [34] reported a weak correlation between higher CSF NfLs and cortical thinning in temporo-parietal areas and hippocampus. Using blood NfLs, Rajan and colleagues [46] documented a negative correlation with whole-brain and hippocampal volume and a positive correlation with third ventricular volume. Asken et al. [32] found that in a sample including AD, MCI patients, and controls, higher plasma NfLs were associated with lower parietal GM volume, while in a group including AD, MCI, and subjective cognitive decline (SCD) patients, there was a positive association with medial temporal lobe atrophy (MTA) score [51]. Oeckl and colleagues [58] worked on data from the German FTLD Consortium, including patients with a clinical diagnosis of AD. They performed atlas-based volumetry and found a negative association between serum NfLs and GM volume in frontal, temporal, and parietal areas, insular cortex, cingulate gyrus, striatum, hippocampus, and amygdala, mostly on the left. Conversely, in the AddNeuroMed study [49] voxelwise association between baseline plasma NfLs and longitudinal changes in GM volume in AD, MCI patients, and controls did not have significant results.

Moscoso et al. [44] distinguished ADNI patients with Aβ+ and Aβ- and found a positive association between plasma NfLs and progression of atrophy in the dorsao-frontal lobe in Aβ- subjects, and in AD-vulnerable regions (i.e., entorhinal, fusiform, inferior temporal, and middle temporal cortices) in Aβ+ participants. When separating AD and MCI patients from controls, irrespectively from biomarkers, the same association was seen with baseline and longitudinal atrophy in parieto-temporal areas in AD and MCI patients, and with longitudinal frontal atrophy in controls.

Both Pereira et al. [45] and Kang et al. [40] divided ADNI patients by clinical phenotype and Aβ status. In healthy controls, CSF NfLs were negatively associated with cortical thickness in the right cuneus [45] and GM volume in the lateral orbitofrontal cortex [40] in Aβ- patients, and with cortical thickness in the left frontal pars triangularis, left temporal pole, right precentral, and right superior temporal gyri [45], and GM volume in the cingulate cortex [40] in Aβ+ individuals. In MCI Aβ- patients, CSF NfLs correlated to cortical thickness of the right precuneus and the left middle frontal, left inferior temporal, and right lingual gyri; moreover, plasma NfLs were associated with cortical thickness of the left middle frontal, lingual, and inferior temporal gyri, right posterior cingulate, insula, inferior parietal, and middle frontal gyri [45], and with GM volume of the insular cortex [40]. In MCI Aβ+ patients, CSF NfLs were associated with cortical thickness and GM volume in the bilateral orbitofrontal cortex and precuneus [40,45], with cortical thickness in the left fusiform, right entorhinal, and left postcentral gyri [45], and with GM volume in the lateral temporal and posterior cingulate cortex, hippocampus, and thalamus [40]. Plasma NfLs were associated with cortical thickness of bilateral fronto-parieto-temporo-occipital areas (mostly of the left precuneus and right superior parietal gyrus) [45], and with GM volume in the lateral temporal cortex and right hippocampus [40]. Finally, in AD patients (all Aβ+), CSF NfLs were associated with cortical thickness in the left middle temporal, left orbitofrontal, left inferior parietal, right supramarginal, and right superior frontal gyri, and plasma NfLs with cortical thickness in the right precuneus, superior temporal, supramarginal, and middle frontal gyri [45]. Both CSF and plasma NfLs correlated to GM volume of dorso-lateral frontal, lateral temporal, and medial frontal cortices, angular gyrus, precuneus and posterior cingulate cortex, hippocampus, and cerebellum [40]. In Pereira et al. [45], some significant correlations also emerged with subcortical structures such as nucleus accumbens, hippocampus, and amygdala in controls and MCI patients.

Some studies focused specifically on AD patients with a biomarker-based diagnosis. Among them, Contador et al. [37] conducted a study in a small sample of early-onset AD patients (*n* = 12) with CSF positivity for AD biomarkers and found that higher CSF NfL levels correlated with higher volume of the left lateral ventricle. Only considering AD patients together with controls (*n* = 19), they found a correlation between baseline CSF NfLs and longitudinal cortical thinning and subcortical structure volume reduction across the all brain. Alcolea and colleagues [55] reported a negative association between CSF NfLs and cortical thickness in the left lateral temporal lobe in 33 AD patients. Conversely, a larger study on 112 AD patients with positive CSF biomarkers failed to find a significant association between CSF NfLs and MTA and posterior atrophy scores [41].

Only a few studies have focused on microstructural changes in WM or metabolic imaging. Falgàs et al. [56] showed, in 64 early-onset AD patients, a negative correlation between CSF NfLs and fractional anisotropy (FA) in the corticospinal tract and uncinate, inferior-longitudinal, and inferior fronto-occipital fasciculi. Across all subjects and cognitively unimpaired controls, a significant association between plasma NfLs and hippocampal microstructures (cornus ammonis 4 and dentate gyrus) was found, whereas no association emerged in AD or MCI patients alone [48]. Both fluorodeoxyglucose (FDG)-PET hypometabolism [9,35,42,43,44] and amyloid-PET burden [38,51,76] were positively associated with CSF and plasma NfLs in AD and MCI patients, and controls, analyzed together or in separate groups, both at baseline and longitudinally. Finally, an association between plasma NfLs and tau-PET in fronto-temporal regions has been reported in a group of MCI and AD patients, while the amyloid-PET load in fronto-parieto-temporal areas showed a negative correlation with plasma NfLs in cognitively unimpaired individuals [33].

Some interest has also been placed on white matter hyperintensities (WMHs), as they can represent a type of WM involvement that is due to vascular damage but adds to the neurodegenerative process in causing cognitive impairment. WMH volume was positively correlated with baseline and longitudinal CSF and plasma NfL levels in AD, MCI [46,54], and SCD [52] patients. Moreover, higher CSF NfLs were found in AD patients with subcortical vascular damage than in AD patients without it [39,50]. Interestingly, Chong et al. [36] showed, using a linear regression model of a group of patients with AD, MCI, VaD, and healthy subjects, that both WMHs and brain atrophy (assessed as MTA score and hippocampal atrophy) were independently associated with plasma NfL levels.

Studies of familial AD patients, including APP, PSEN1, and PSEN2 mutation carriers, showed a significant negative correlation between serum NfLs and whole-brain and ventricular volume, both at baseline and longitudinally, and with hippocampal volume at baseline [53]. When studying WM in AD mutation carriers [47], serum NfLs correlated with baseline WMH volume, diffusion tensor imaging (DTI) metrics (i.e., FA, mean, axial, and radial diffusivity—MD, AxD, RD) in the posterior corpus callosum, forceps, frontal aslant tracs, superior longitudinal, inferior-longitudinal, and uncinate fasciculi. Serum NfLs also correlated with DTI metrics in the corpus callosum over time, while this association was missing in non-carriers. After dividing mutation carriers based on clinical symptoms (i.e., presymptomatic versus symptomatic patients), the association between NfLs and WM damage was stronger in symptomatic or presymptomatic individuals close to the onset of cognitive symptoms.

### 3.3. Frontotemporal Dementia Spectrum

Most of the studies investigating the association between NfLs and neuroimaging measures in the FTD spectrum have used VBM or region of interest (ROI) analysis, focusing on GM reduction in fronto-temporal areas.

In the first report, Scherling and colleagues [18] studied the association between CSF NfLs and different clinical measures of disease severity in 66 FTD patients, including bvFTD, svPPA, and non-fluent variant primary progressive aphasia (nfvPPA). They found a negative correlation between CSF NfLs and GM volume in frontal, temporal, parietal, occipital, and cingulate cortices, mostly in the left hemisphere. A smaller study of 46 FTD subjects, of whom 29 had a longitudinal MRI scan, only found an association between serum NfLs and frontal lobe GM atrophy rate, with no significant correlations at baseline [19]. Another study, which separately analyzed the three phenotypes, found a correlation between CSF NfLs and basal fronto-temporal volume in bvFTD patients, and an association with the rate of fronto-temporal volume reduction in bvFTD and nfvPPA patients. Patients with svPPA only showed an association with the right temporal volume with a trend toward significance [63].

Focusing on bvFTD patients only, Steinacker and colleagues [73] found a negative correlation between serum NfLs and GM in frontal and—more weakly—temporal lobes. The same correlation was also found with striatal and limbic system regions, with WM volume in the frontal lobe, and with longitudinal frontal GM, while parietal or occipital regions did not correlate with NfLs. Similarly, Oeckl et al. [58] reported some results from the German FTLD Consortium, among which bvFTD patients showed a negative association between serum NfLs and GM volume in the frontal cortex and striatum.

Steinacker et al. [72] also studied 22 PPA patients longitudinally, finding that a higher increase in serum NfLs over time positively correlated with more pronounced atrophy progression in the bilateral frontal lobes, particularly in the right middle frontal gyrus and in the left gyrus rectus in all PPA patients, and the right middle frontal gyrus in nfvPPA and svPPA separately. Baseline analysis and correlations between NfLs at baseline and longitudinal imaging did not reach significance or survive correction for multiple comparisons. In svPPA only, Meeters et al. [66] found a correlation between CSF NfLs and GM atrophy at baseline in the parahippocampal gyrus of the more atrophic side, and a trend of association in the medial and inferior temporal gyri in 87 svPPA patients – 65 left-dominants, and 22 right-dominants. Oeckl and colleagues [58] reported an association between higher serum NfLs and cross-sectional GM atrophy in fronto-temporo-occipital areas in svPPA, and in the hippocampus and right amygdala in nfvPPA.

As for the extrapyramidal phenotypes, which are currently included in the FTD spectrum, few studies investigated the relationship between NfLs and PSP, finding an association between NfLs and atrophy both at baseline and over time, mostly in the superior cerebellar peduncle [68,69]. Surprisingly, Painous et al. [67] showed a correlation between higher CSF NfL levels and more atrophy in midbrain and pons in PSP subjects and a correlation with more volume of the same areas in CBS individuals. The possible interpretation provided by the authors is that the results could reflect an initial inflammation process that precedes atrophy. Conversely, another study demonstrated an association between serum NfLs and atrophy in the left putamen and supramarginal gyrus in CBS patients, and no significant interaction in PSP subjects [58].

Fewer studies examined this relationship in terms of cortical thickness. The first one included 132 FTD patients, including extrapyramidal phenotypes, and found an inverse correlation between serum NfL levels and cortical thickness of the prefrontal, temporal, and parietal regions, mainly in the dorso-lateral prefrontal cortex and on the left side [59]. Similar results have been shown with CSF NfLs, in areas of the bilateral frontal lobe such as the left pars opercularis, pars triangularis, middle and superior frontal, and precentral gyri, and the right prefrontal and orbitofrontal cortices [56,71], and in the temporal and parietal lobes [55]. Conversely, Illán-Gala and colleagues [61] investigated the same association in FTD and ALS patients and did not report significant results. Interestingly, a study that compared AD and FTD spectrum showed that plasma NfLs strongly correlated with cortical thickness in frontal regions in the FTD spectrum, and in the right lateral temporal, right inferior parietal, and left superior frontal cortices in the AD group [57]. Another innovative method for studying the cerebral cortex is cortical MD, which reflects microstructural disorganization and disruption of cellular membranes inside the cortex being an earlier marker of macroscopic cortical changes. A study that evaluated both analyses in bvFTD patients revealed that CSF NfL levels were associated with cortical thickness and – to a greater extent – cortical MD in dorso-lateral and medial prefrontal, temporal, and parietal regions, suggesting that cortical diffusivity could be a more sensitive method for detecting cortical neurodegeneration [62].

As for WM microstructural integrity, Spotorno et al. [71] showed a negative correlation between plasma NfLs and FA in the uncinate and inferior fronto-occipital fasciculi, the anterior thalamic radiation, the corpus callosum, the left corticospinal tract, and the cerebral peduncle in bvFTD patients, while Falgàs and colleagues [56] reported an association between CSF NfLs and FA in the forceps minor, anterior thalamic radiation, cingulum, and left superior longitudinal fasciculus in all FTD patients. Longitudinally, it has been shown that higher baseline CSF NfLs predict a faster rate of decline in FA in frontotemporal areas in bvFTD and nfvPPA, and show a trend toward significance in the right uncinated fasciculus and the genu of corpus callosum in svPPA [63].

We identified only six studies that focused specifically on genetic FTD, including C9ORF72 repeat expansion, GRN, and MAPT mutation carriers. Meeters et al. [64] found a negative correlation between CSF NfLs and GM volume of the frontal, temporal, parietal, cingulate, and insular cortices in 165 symptomatic and asymptomatic mutation carriers. In the subgroup of symptomatic carriers, the same correlation was limited to frontal and insular cortices, while in the presymptomatic carriers, negative correlations were found for frontal, temporal, and parietal cortices. In a limited subgroup of carriers with a follow-up MRI scan (17 subjects), a significant correlation between CSF NfLs and the annual rate of atrophy was found again for all areas except for the occipital cortex. The same group, three years later, expanded the longitudinal data [20] by following 258 symptomatic, presymptomatic – converters or non-converters – mutation carriers, and non-carriers for 2 years. They demonstrated, across all groups, an association between the rate of serum NfL increase and volume reduction in frontal and temporal lobes, insula, cingulate gyrus, hippocampus, putamen, amygdala, and cerebellum. A more recent study supports these findings, showing a correlation between baseline plasma NfLs and fronto-temporal brain atrophy on a 2-year follow-up, in asymptomatic and symptomatic mutation carriers [70]. Specifically, in C9ORF72 expansion carriers only, both symptomatic and presymptomatic patients showed a correlation between higher CSF NfL levels and lower frontal GM volumes in an ROI analysis, including 12 cortical areas and subcortical structures, and between higher NfL levels and lower GM volumes in fronto-temporo-parietal structures in a whole-brain VBM analysis [65]. Another work, which compared clinical features of C9ORF72 carriers and non-carriers, found a negative correlation between serum NfL concentration and mean cortical thickness at baseline and GM volume progression in the frontal cortex, thalamus, caudate, pallidum, and putamen nuclei over time within the entire group of patients, whereas they did not report differences in this association between carriers and non-carriers [60]. Only one study [74] exclusively investigated GRN mutation carriers, focusing on WMHs, since they are more commonly associated with this genotype: in a group of 133 GRN carriers, plasma NfLs were associated with both WMH load at baseline and WMH increase longitudinally.

### 3.4. Mild Cognitive Impairment and Subjective Cognitive Decline

As for AD, the literature on MCI patients is often based on large cohorts of subjects, where group characterization is based mostly on clinical grounds and without a clear biomarker-based diagnosis. For example, a significant correlation between CSF and plasma NfL levels and hippocampal volume and global cortical thickness, at baseline and over time, was found in ADNI MCI patients [54,78], as well as an association with perirhinal GM volume longitudinally [87] and with the left inferior frontal and medial temporal gyri volume in amnestic MCI only [85].

Similar results have been found in studies that analyzed together MCI and cognitively unimpaired subjects, which found a correlation between baseline CSF NfL levels and baseline atrophy in fronto-parieto-temporal and cingulate cortices [82], and between baseline plasma and CSF NfL levels and worsening of hippocampal volume and global atrophy longitudinally [80]. Conversely, when comparing MCI and healthy subjects, the latter presented a more widespread pattern of relationship between serum NfLs and GM volume, while in the former, the association involved specific areas such as calcarine fissure and cortex, lingual gyrus, nucleus accumbens, hippocampus, and putamen [77].

Huang and colleagues [76] focused on SCD patients, analyzed in a single group with healthy controls, and again reported a negative correlation between plasma NfLs and hippocampal volume and global cortical thickness.

Studies investigating WM integrity in MCI patients suggested an association between CSF and plasma NfLs and all measures of WM damage, including negative associations with FA in the corpus callosum, both at baseline and over time [78,80], in superior corona radiata and posterior thalamic radiation, and positive associations with MD and AxD in the anterior corona radiata, and with RD in the striatum [75,81]. Moreover, these associations proved stronger among subjects with higher total tau and lower Aβ-42 [81]. Nabizadeh et al. [83] divided MCI patients based on ApoE status, finding a correlation with plasma NfLs in the internal capsule, corona radiate, fornix, corpus callosum, inferior fronto-occipital fasciculus, and sagittal stratum in ApoE ε4 carriers, and in corona radiate, internal capsule, hippocampal cingulum, and uncinate fasciculus in non-carriers.

As concerns PET-based studies, an association between plasma and CSF NfLs and both FDG-PET hypometabolism and amyloid-PET burden has been reported, at baseline and longitudinally [75,80], not only in MCI but also in SCD [76] patients.

Finally, NfL levels in MCI patients have also been examined in relation to WMHs and their progression over time, with a positive correlation being shown both at baseline and longitudinally [79,84,86].

### 3.5. Lewy Body Dementia

No studies on LBD directly searched for an association between NfL levels and MRI measures. Likewise, information is lacking on Parkinson’s disease with dementia (PDD) since the few studies that reported a correlation between NfLs and either GM atrophy or WM integrity included patients with early PD diagnosis and without cognitive impairment [88,89,90,91,92].

## 4. Discussion

The present review provides an overview of the current knowledge on the correlation between NfLs and imaging measures in neurodegenerative dementias. The vast majority of included studies reported a significant positive correlation between higher NfL levels and greater diffuse GM atrophy, WM microstructural disruption, glucose hypometabolism, and specific protein accumulation, both cross-sectionally and longitudinally. This supports the idea that increased NfL levels accurately reflect the presence, extent, and speed of the neurodegenerative process, confirming their role as a biomarker of disease severity and prognosis in neurodegenerative dementias. For the same reasons, NfLs may also help as biomarkers of treatment response in clinical trials, as suggested by recent studies such as that by Pontecorvo et al. [93]. Moreover, their association not only with cortical GM but also with WM, subcortical structures volumes, and WMHs suggests that areas rich in large-caliber myelinated axons exert a more pronounced influence on NfL release.

Even though the number of studies that have explored this topic is large, their results are quite inconsistent and difficult to summarize due to several limitations.

Firstly, the techniques and type of sample used (i.e., CSF, serum, plasma) to measure NfLs were different among studies. Although there is evidence of a high correlation and consistency between them, this may be a source of difference in the results. For example, Chong et al. [36] and Verberk et al. [51] found a significant correlation between plasma NfLs and MTA score, while the same association has not been found by Mao et al. [41] when measuring NfLs in CSF.

Moreover, some studies performed a logarithmic transformation of NfL values to obtain a normal distribution, while other studies used raw values.

The imaging measures investigated vary greatly between studies, particularly regarding structural analysis of brain volume that has been assessed with cortical thickness or VBM, whole-brain, or ROI-based analysis. Cortical thickness provides information about the thickness of the cortex across the entire brain, while VBM analyzed differences in GM density at a voxel level. The analysis can be conducted on the whole brain or specific regions defined a priori. Consequently, the output and the related results may conflict or be slightly different, as in the case of studies of Pereira et al. [45] and Kang et al. [40], where the first assessed cortical thickness while the second performed a VBM analysis.

Another limitation is that most of the included studies primarily focused on the role of NfLs and other fluid biomarkers as diagnostic and prognostic markers in neurodegenerative dementias, and principally analyzed the differences between diagnostic groups or their changes along disease progression. Consequently, the correlations with imaging measures were only assessed as a secondary outcome or reported briefly.

Additionally, study populations were heterogeneous, including different clinical phenotypes (e.g., behavioral and linguistic variants of FTD, extrapyramidal phenotypes of FTD) at different clinical stages (e.g., SCD, MCI, dementia), and frequently the correlations between NfLs and imaging were analyzed across the whole groups. Moreover, cognitively unimpaired individuals, who were included as controls, were often analyzed together with patients.

Among studies on AD, several authors classified patients only on clinical criteria, without specific biomarker confirmation. MCI and SCD patients were also clinically classified, independently from biomarkers and follow-up information, so that different underlying pathologies could be analyzed together.

Finally, the covariates selected for the association analysis were different among studies. In particular, only a few studies analyzed the correlation with imaging measures, including age as a covariate to be accounted for, even though it is widely recognized that age has an influence on NfL levels. For example, Walsh et al. [52] found a significant association between WMHs and NfLs, but this association became substantially weaker when age was introduced as a covariate, probably suggesting that age had the strongest impact. Moreover, Pereira et al. [45] and Kang et al. [40] both adjusted the analysis for age, but Kang et al. [40] also adjusted for ApoE status, education, CSF phosphorylated tau, and time between MRI scans, finding some differences in the extent of associations.

For all these limitations, disease-specific patterns of spatial correlations between NfLs and imaging measures are difficult to identify, particularly in AD and FTD where studies are more numerous. Overall, it appears that correlations were stronger in temporo-parietal areas in AD individuals and fronto-temporal areas in FTD individuals. This difference, which may seem trivial and predictable, actually reflects the non-specific nature of NfLs as a biomarker. In fact, the pattern of correlation with specific focal brain areas was probably driven by the greater atrophy in those regions. The same applies to hypometabolism or WM microstructural integrity. In other words, NfL levels appear to be mostly influenced by the severity of the neurodegenerative process and the degree of brain atrophy, rather than a specific spatial distribution.

## 5. Conclusions

This scoping review investigated the association between NfLs and imaging measures in neurodegenerative dementias, showing a strong correlation between higher NfL levels and GM atrophy as well as WM damage and hypometabolism. This confirms that NfLs accurately reflect neurodegeneration and represent a useful biomarker of disease severity and prognosis, but that they are not disease- or region-specific.

## Figures and Tables

**Table 1:** Key findings of identified studies. Studies are presented in alphabetical order according to first authors’ names.

Study	Numbers	Imaging Measures	Significant Key Findings		Study Design	Assay
**Alzheimer’s Disease**						
Asken et al. (2022) [32]	Cohort 1: 11 MCI, 39 HC Cohort 2: 21 AD, 18 MCI, 32 HC	GM, WM	Plasma NfLs	All subjects (cohort 2): ↓ cross-sectional parietal GM volume	Cross-sectional	Simoa (plasma)
Benedet et al. (2020) [33]	767 MCI/AD, 382 HC from ADNI database, 42 MCI/AD, 74 HC from the TRIAD database	GM, WM, amy-PET, tau-PET	Plasma NfLs	MCI/AD: -↓ cross-sectional fronto-temporal and longitudinal temporal GM volume ↓ cross-sectional and longitudinal whole-brain WM volume -↑ cross-sectional fronto-temporal tau burden HC: -↓ cross-sectional frontal and hippocampal and longitudinal fronto-temporal GM volume -↓ cross-sectional fronto-parietal and longitudinal superior periventricular WM volume -↑ cross-sectional fronto-parieto-temporal amyloid burden	Cross-sectional and longitudinal	Simoa (plasma)
Boerwinkle et al. (2021) [34]	371 AD, MCI, and HC (group numbers not specified)	CTh	CSF NfLs	All subjects: ↓ cross-sectional temporo-parietal and hippocampal CTh AD: ↓ cross-sectional temporo-parietal and hippocampal CTh	Cross-sectional	ELISA (CSF)
Chen et al. (2021) [35]	57 AD, 120 MCI, 67 HC from ADNI database	GM, ventricular volume, FDG-PET	CSF NfLs	MCI: ↓ cross-sectional hippocampal volume; ↑ cross-sectional ventricular volume AD: -↑ cross-sectional ventricular volume -↑ cross-sectional FDG hypometabolism HC: -↓ cross-sectional hippocampal volume; ↑ cross-sectional ventricular volume; -↑ cross-sectional FDG hypometabolism	Cross-sectional	Simoa (plasma), ELISA (CSF)
			Plasma NfLs	MCI: -↓ cross-sectional hippocampal volume; ↑ cross-sectional ventricular volume -↑ cross-sectional FDG hypometabolism AD: ↓ cross-sectional hippocampal volume; ↑ cross-sectional ventricular volume HC: -↓ cross-sectional hippocampal volume; ↑ cross-sectional ventricular volume -↑ cross-sectional FDG hypometabolism		
Chong et al. (2023) [36]	44 AD, 99 MCI, 22 VaD, 43 HC	GM, MTA score, WMHs, amy-PET	Plasma NfLs	All subjects: -↓ cross-sectional hippocampal volume; ↑ cross-sectional MTA score -↑ cross-sectional WMH volume	Cross-sectional	Simoa (plasma)
Contador et al. (2021) [37]	12 early onset AD, 19 HC	CTh, subcortical GM, ventricular volume	CSF NfLs	All subjects: ↓ longitudinal CTh and subcortical structures GM volume AD: ↑ cross-sectional left lateral ventricle volume	Cross-sectional and longitudinal	ELISA (CSF)
Dhiman et al. (2020) [38]	28 AD, 34 MCI, 159 HC from the AIBL database (179 cross-sectional MRI, 195 cross-sectional amy-PET, 118 longitudinal amy-PET)	GM, WM, amy-PET	CSF NfLs	All subjects: -↓ cross-sectional whole-brain and hippocampal volume -↑ cross-sectional amyloid burden	Cross-sectional and longitudinal	ELISA (CSF)
Elahi et al. (2020) [39]	63 AD, 33 HC	WMHs	Plasma NfLs	All subjects: ↑ cross-sectional WMH volume Higher in AD with high WMHs burden than in AD with lower WMHs burden	Cross-sectional	Simoa (plasma)
Kang et al. (2021) [40]	73 AD, 160 MCI (112 Aβ+, 48 Aβ-), 83 HC (17 Aβ+, 66 Aβ-) from ADNI database	GM	CSF NfLs	MCI Aβ+: ↓ cross-sectional fronto-temporo-parietal, hippocampal and thalamic GM volume AD: ↓ cross-sectional fronto-temporo-occipito-parietal, hippocampal and cerebellar GM volume HC Aβ+: ↓ cross-sectional cingulate GM volume HC Aβ-: ↓ cross-sectional orbitofrontal GM volume	Cross-sectional	Simoa (plasma), ELISA (CSF)
			Plasma NfLs	MCI Aβ+: ↓ cross-sectional temporal and hippocampal GM volume MCI Aβ-: ↓ cross-sectional insular GM volume AD: ↓ cross-sectional fronto-temporo-occipito-parietal, hippocampal and cerebellar GM volume		
Mao et al. (2021) [41]	112 AD, 30 HC	MTA, PA scores	CSF NfLs	No significant associations	Cross-sectional	ELISA (CSF)
Mattson et al. (2016) [42]	93 AD, 187 MCI, 109 HC from ADNI database	GM, ventricular volume, FDG-PET	CSF NfLs	Aβ+: -↓ cross-sectional hippocampal volume; ↑ cross-sectional and longitudinal ventricular volume -↑ longitudinal FDG hypometabolism Aβ-: -↓ cross-sectional and longitudinal hippocampal volume; ↑ cross-sectional and longitudinal ventricular volume -↑ longitudinal FDG hypometabolism	Cross-sectional and longitudinal	ELISA (CSF)
Mattson et al. (2017) [9]	180 AD, 197 MCI, 193 HC from ADNI database	CTh, GM, ventricular volume, WMHs, FDG-PET	Plasma NfLs	All subjects: -↓ cross-sectional and longitudinal hippocampal volume and occipito-temporal CTh; ↑ cross-sectional and longitudinal ventricular volume -↑ longitudinal FDG hypometabolism	Cross-sectional and longitudinal	Simoa (plasma), ELISA (CSF)
Mattsson et al. (2019) [43]	327 AD, 855 MCI, 1583 HC from ADNI database	CTh, GM, WMHs, FDG-PET	Baseline plasma NfLs	All subjects: -↓ cross-sectional entorhinal cortex and hippocampal volume; ↑ cross-sectional ventricular volume -↑ cross-sectional FDG hypometabolism	Cross-sectional and longitudinal	Simoa (plasma)
			Longitudinal plasma NfLs	All subjects: -↓ cross-sectional entorhinal cortex and hippocampal volume; ↑ cross-sectional ventricular volume -↑ cross-sectional FDG hypometabolism MCI: ↓ longitudinal entorhinal cortex and hippocampal volume; ↑ longitudinal ventricular volume AD: ↑ longitudinal ventricular volume HC: ↓ longitudinal entorhinal cortex and hippocampal volume; ↑ longitudinal ventricular volume		
Moscoso et al. (2021) [44]	198 AD, 537 MCI, 378 HC from ADNI database (554 Aβ+, 559 Aβ-)	GM, FDG-PET	Plasma NfLs	All subjects: ↑ longitudinal FDG hypometabolism All subjects Aβ-: ↓ longitudinal dorso-frontal GM volume All subjects Aβ+: -↓ longitudinal temporal GM volume -↑ cross-sectional FDG hypometabolism MCI/AD: -↓ cross-sectional temporo-parietal GM volume; ↓ longitudinal frontal GM volume -↑ cross-sectional FDG hypometabolism HC: -↓ longitudinal frontal GM volume -↑ cross-sectional fronto-temporal FDG hypometabolism HC Aβ-: ↑ cross-sectional FDG hypometabolism	Cross-sectional and longitudinal	Simoa (plasma)
Pereira et al. (2017) [45]	65 AD, 145 MCI (109 Aβ+, 36 Aβ-), 94 HC (37 Aβ+, 57 Aβ-) from ADNI database	CTh, subcortical GM	CSF NfLs	MCI Aβ+: ↓ cross-sectional fronto-temporo-parieto-occipital CTh and putamen GM volume MCI Aβ-: ↓ cross-sectional fronto-temporo-occipital CTh and subcortical structures GM volume AD: ↓ cross-sectional fronto-temporo-parietal CTh HC Aβ+: ↓ cross-sectional fronto-temporal CTh HC Aβ-: ↓ cross-sectional right cuneus CTh and accumbens GM volume	Cross-sectional	Simoa (plasma), ELISA (CSF)
			Plasma NfLs	MCI Aβ+: ↓ cross-sectional fronto-temporo-parieto-occipital CTh and subcortical structures GM volume MCI Aβ-: ↓ cross-sectional fronto-temporal, parieto-cingulate, and insular CTh and subcortical structures GM volume AD: ↓ cross-sectional fronto-temporo-parietal CTh		
Rajan et al. (2020) [46]	421 AD, 317 MCI, 634 HC (742 cross-sectional MRI, 183 longitudinal MRI)	GM, ventricular volume, WMHs	Blood NfLs	All subjects: -↓ cross-sectional whole-brain and longitudinal hippocampal volume; ↑ cross-sectional 3rd ventricular volume -↑ cross-sectional WMH volume	Cross-sectional and longitudinal	Simoa (blood)
Schultz et al. (2020) [47]	117 familial AD ^1^ (76 asymptomatic carriers, 41 symptomatic carriers), 84 HC	WM	Serum NfLs	Mutation carriers: -↑ cross-sectional WMH volume -↓ cross-sectional posterior corpus callosum, SLF, ILF, UF, forceps, corticospinal and frontal tracts FA and ↑ MD, RD and AxD; ↓ longitudinal corpus callosum FA and ↑ MD, RD and AxD	Cross-sectional and longitudinal	Simoa (serum)
Shahid et al. (2022) [48]	19 AD, 52 MCI, 47 HC	WM	Plasma NfLs	All subjects: ↓ cross-sectional NODDI-derived parameters of WM integrity in CA4-DG HC: ↓ cross-sectional NODDI-derived parameters of WM integrity in CA4-DG	Cross-sectional	Simoa (plasma)
Simrén et al. (2021) [49]	103 AD, 107 MCI, 99 HC	GM	Plasma NfLs	No significant associations	Longitudinal	Simoa (plasma)
Sjögren et al. (2001) [50]	22 AD (9 WMH+, 13 WMH-), 20 HC	WMHs	CSF NfLs	Higher in AD WMH+ patients than AD WMH- and HC	Cross-sectional	ELISA (CSF)
Verberk et al. (2020) [51]	132 AD, 50 MCI, 70 SCD (182 cross-sectional MRI)	MTA score, amy-PET	Plasma NfLs	All subjects: ↑ cross-sectional MTA score and amyloid burden	Cross-sectional	Simoa (plasma)
Walsh et al. (2021) [52]	130 AD, 431 MCI, 103 SCD, 163 HC from ADNI database	WMHs	Plasma NfLs	MCI: ↑ cross-sectional WMH volume AD: ↑ cross-sectional WMH volume SCD: ↑ cross-sectional WMH volume HC: ↑ cross-sectional WMH volume	Cross-sectional	Simoa (plasma)
Weston et al. (2017) [53]	37 familial AD ^1^ (18 symptomatic, 19 asymptomatic), 11 HC (43 cross-sectional MRI, 33 longitudinal MRI)	GM, ventricular volume	Serum NfLs	Mutation carriers: ↓ cross-sectional whole-brain and hippocampal volume; ↓ longitudinal whole-brain volume; ↑ cross-sectional and longitudinal ventricular volume	Cross-sectional and longitudinal	Simoa (serum)
Zetterberg et al. (2016) [54]	95 AD, 192 MCI, 110 HC from ADNI database	GM	CSF NfLs	All subjects: -↓ longitudinal whole-brain and hippocampal volume; ↑ longitudinal ventricular volume -↑ longitudinal WMH volume MCI: -↓ cross-sectional hippocampal volume -↑ cross-sectional WMH volume AD: ↑ cross-sectional WMH volume	Cross-sectional and longitudinal	ELISA (CSF)
**Alzheimer’s Disease and Frontotemporal Dementia**						
Alcolea et al. (2017) [55]	72 AD, 159 FTD, 76 HC (115 cross-sectional MRI)	CTh	CSF NfLs	FTD: ↓ cross-sectional fronto-temporo-parietal CTh AD: ↓ cross-sectional temporo-lateral CTh	Cross-sectional	ELISA (CSF)
Falgàs et al. (2020) [56]	64 early onset AD, 26 FTD, 48 HC	CTh, WM	CSF NfLs	All subjects: ↓ cross-sectional hippocampal CTh FTD: -↓ cross-sectional frontal CTh -↓ cross-sectional forceps minor, anterior thalamic radiation, cingulum and left SLF FA AD: ↓ cross-sectional corticospinal tract, UF, ILF, and IFOF FA	Cross-sectional	ELISA (CSF)
Illán-Gala et al. (2021) [57]	167 FTD spectrum (43 bvFTD, 28 nfvPPA, 18 svPPA, 36 PSP, 32 CBS, 10 FTD-ALS), 43 AD, 55 HC (240 cross-sectional MRI)	CTh	Plasma NfLs	FTD spectrum: ↓ cross-sectional frontal CTh AD: ↓ cross-sectional fronto-temporo-parietal CTh	Cross-sectional	Simoa (plasma)
Oeckl et al. (2023) [58]	74 AD, 81 bvFTD, 41 svPPA, 55 nfvPPA, 25 lvPPA, 42 PSP, 25 CBS, 31 HC	GM	Serum NfLs	All subjects: ↓ cross-sectional fronto-temporo-parietal, cingulate, insular, hippocampal, and subcortical structures GM volume AD: ↓ cross-sectional fronto-temporo-parietal, cingulate, insular, hippocampal, and subcortical structures GM volume bvFTD: ↓ cross-sectional frontal and striatum GM volume svPPA: ↓ cross-sectional fronto-temporal and occipital GM volume nfvPPA: ↓ cross-sectional hippocampus and right amygdala GM volume CBS: ↓ cross-sectional left putamen and supramarginal gyrus GM volume	Cross-sectional	ELLA (serum)
**Frontotemporal Dementia**						
Benussi et al. (2020) [59]	134 bvFTD, 48 nfvPPA, 27 svPPA, 51 CBS, 31 PSP, 63 AD, 63 HC (132 cross-sectional MRI)	CTh	Serum NfLs	FTD spectrum: ↓ cross-sectional prefrontal and temporo-parietal CTh	Cross-sectional	Simoa (serum)
Cajanus et al. (2020) [60]	26 FTD C9ORF72+, 52 FTD C9ORF72 (41 cross-sectional MRI, 11 longitudinal MRI)	CTh, subcortical GM	Serum NfLs	All subjects: ↓ cross-sectional median CTh and longitudinal frontal and subcortical structures GM volume	Cross-sectional and longitudinal	Simoa (serum)
Illán-Gala et al. (2018) [61]	86 FTD, 38 ALS, 49 HC (70 cross-sectional MRI)	CTh	CSF NfLs	No significant associations	Cross-sectional	ELISA (CSF)
Illán-Gala et al. (2019) [62]	70 bvFTD, 78 HC (32 available CSF)	CTh, WM	CSF NfLs	bvFTD: -↓ cross-sectional dorsolateral and medial prefrontal CTh -↑ cross-sectional fronto-temporo-parietal cortical MD	Cross-sectional	ELISA (CSF)
Ljubenkov et al. (2018) [63]	40 bvFTD, 24 svPPA, 26 nfvPPA, 49 HC (81 cross-sectional MRI)	GM, WM	CSF NfLs	bvFTD: -↓ cross-sectional and longitudinal fronto-temporal GM volume -↓ longitudinal fronto-temporal FA nfvPPA: -↓ longitudinal fronto-temporal GM volume -↓ longitudinal fronto-temporal FA	Cross-sectional and longitudinal	ELISA (CSF)
Meeter et al. (2016) [64]	165 genetic FTD ^2^ (102 symptomatic carriers, 63 asymptomatic carriers), 73 HC (101 cross-sectional MRI, 22 longitudinal MRI)	GM	CSF NfLs	All carriers: ↓ cross-sectional and longitudinal whole-brain and fronto-temporo-parietal, cingulate, and insular GM volume Symptomatic carriers: ↓ cross-sectional whole-brain, frontal, and insular GM volume Asymptomatic carriers: ↓ cross-sectional whole-brain and fronto-temporo-parietal GM volume	Cross-sectional and longitudinal	ECLIA (serum), ELISA (CSF)
Meeter et al. (2018) [65]	89 C9ORF72 FTD (64 symptomatic, 25 asymptomatic), 12 HC (63 cross-sectional MRI)	GM	CSF NfLs	All carriers: ↓ cross-sectional frontal, insular, cingulate, and subcortical structures GM volume Symptomatic: ↓ cross-sectional frontal GM volume	Cross-sectional	ELISA (CSF)
Meeter et al. (2019) [66]	162 svPPA (87 cross-sectional MRI, 37 longitudinal MRI) and 65 HC	GM	CSF NfLs	svPPA: ↓ parahippocampal gyrus (of the dominant atrophic side) GM volume	Cross-sectional and longitudinal	ELISA (CSF)
Painous et al. (2023) [67]	21 PSP, 14 CBS, 26 MSA, 12 PD, 11 HC	Brainstem volume	CSF NfLs	PSP: ↓ cross-sectional midbrain and pons volume CBS: ↑ cross-sectional midbrain and pons volume	Cross-sectional	ELISA (CSF)
Rohrer et al. (2016) [19]	67 FTD, HC 28 (46 cross-sectional MRI, 29 longitudinal MRI)	GM	Serum NfLs	FTD: ↓ longitudinal frontal GM volume	Cross-sectional and longitudinal	Simoa (serum)
Rojas et al. (2016) [68]	147 PSP (124 longitudinal MRI)	GM	Plasma NfLs	Patients with higher levels at baseline (≥37.6 pg/mL): greater reduction in whole-brain, midbrain, and superior cerebellar peduncle GM volume and greater ventricle expansion than patients with lower levels	Longitudinal	Simoa (plasma), ELISA (CSF)
Rojas et al. (2018) [69]	50 PSP with CSF (46 longitudinal MRI), 141 PSP with plasma (127 longitudinal MRI)	Brainstem volume	CSF NfLs	PSP: ↓ cross-sectional and longitudinal superior cerebellar peduncle volume	Cross-sectional and longitudinal	Simoa (plasma), ELISA (CSF)
Rojas et al. (2021) [70]	187 genetic FTD ^2^ (95 symptomatic carriers, 92 asymptomatic carriers), 90 HC	GM	Plasma NfLs	All carriers: ↓ longitudinal fronto-temporal GM volume	Cross-sectional and longitudinal	Simoa (plasma, CSF)
Scherling et al. (2014) [18]	79 FTD, 50 AD, 22 PSP, 17 CBS, 6 PD, 47 HC (66 cross-sectional MRI in the FTD group)	GM	CSF NfLs	FTD: ↓ cross-sectional fronto-temporo-occipital and parieto-cingulate GM volume	Cross-sectional	ELISA (CSF)
Spotorno et al. (2020) [71]	20 bvFTD, 22 HC	CTh, WM	Plasma NfLs	bvFTD: -↓ cross-sectional frontal CTh -↑ cross-sectional UF, IFOF, anterior thalamic radiation, corpus callosum, left corticospinal tract and cerebral peduncle FA	Cross-sectional	Simoa (plasma)
Steinacker et al. (2017) [72]	99 PPA, 35 HC (42 cross-sectional MRI, 33 longitudinal MRI)	GM	Longitudinal serum NfLs	All PPA: ↓ longitudinal bilateral frontal GM volume nfvPPA: ↓ longitudinal right middle frontal gyrus GM volume svPPA: ↓ longitudinal right middle frontal gyrus GM volume	Cross-sectional and longitudinal	ECLIA (serum), ELISA (CSF)
Steinacker et al. (2018) [73]	74 bvFTD, 26 AD, 17 MCI, 15 HC (71 cross-sectional MRI, longitudinal not specified)	GM, WM	Serum NfLs	bvFTD: -↓ cross-sectional frontal, striatum, and right amygdala GM volume; ↓ longitudinal frontal GM volume -↓ cross-sectional frontal WM volume	Cross-sectional and longitudinal	Simoa (serum), ELISA (CSF)
Sudre et al. (2019) [74]	133 GRN FTD (32 symptomatic, 101 asymptomatic), 203 HC (124 longitudinal MRI)	WMHs	Plasma NfLs	All carriers: ↑ cross-sectional and longitudinal WMHs load in the medial region and occipital lobe HC: ↑ longitudinal WMHs load in the medial region	Cross-sectional and longitudinal	Simoa (plasma)
van der Ende et al. (2019) [20]	208 genetic FTD ^2^ (59 symptomatic, 149 asymptomatic), 127 HC (276 cross-sectional MRI, 258 longitudinal MRI)	GM	Longitudinal serum NfLs	All subjects: ↓ cross-sectional whole-brain, frontal, insular, cingulate, and temporal GM volume; ↓ longitudinal whole-brain, frontal, insular, cingulate, temporal, subcortical structure and cerebellar GM volume	Cross-sectional and longitudinal	Simoa (serum)
**Mild Cognitive Impairment**						
Andersson et al. (2020) [75]	113 AD, 227 MCI, 478 HC (cross-sectional MRI and amy-PET in MCI and HC only)	WM, amy-PET	CSF NfLs	All subjects: -↓ cross-sectional FA and ↑ MD in all WM tracts -↑ cross-sectional fronto-temporal, parieto-cingulate, and occipital amyloid burden MCI: ↓ cross-sectional FA and ↑ MD in all WM tracts HC: ↓ cross-sectional FA and ↑ MD in all WM tracts	Cross-sectional	Simoa (plasma), ELISA (CSF)
			Plasma NfLs	MCI: ↓ cross-sectional FA in all WM tracts		
Huang et al. (2022) [76]	111 SCD, 123 HC	CTh, GM, amy-PET	Plasma NfLs	All subjects: -↓ cross-sectional hippocampal volume and mean CTh -↑ cross-sectional amyloid burden	Cross-sectional	Simoa (plasma)
Lee et al. (2022) [77]	53 MCI, 146 HC	CTh	Serum NfLs	All subjects: ↓ cross-sectional whole-brain and parieto-temporo-occipital CTh MCI: ↓ cross-sectional calcarine fissure and cortex, lingual gyrus, nucleus accumbens, hippocampal and putamen CTh HC: ↓ cross-sectional whole-brain and parieto-temporo-occipital CTh	Cross-sectional	Simoa (serum)
Marks et al. (2021) [78]	Mayo cohort: 131 MCI, 864 HC ADNI cohort: 197 MCI, 190 HC	CTh, GM, WM	Plasma NfLs	All subjects (Mayo cohort): -↓ cross-sectional fronto-temporo-parieto-occipital CTh; ↓ longitudinal temporal CTh and hippocampal volume -↓ longitudinal corpus callosum FA All subjects (ADNI cohort): ↓ cross-sectional and longitudinal hippocampal volume Aβ+ (Mayo cohort): ↓ cross-sectional corpus callosum FA	Cross-sectional and longitudinal	Simoa (plasma)
Meeker et al. (2022) [79]	71 MCI, 348 HC	GM, WM, WMHs	CSF NfLs	All subjects: -↓ cross-sectional GM volume -↑ WMH volume (survives when accounting for GM, WM, and WMHs at the same time)	Cross-sectional	ELISA (CSF)
Mielke et al. (2019) [80]	15 MCI, 64 HC	CTh, GM, WM, FDG-PET, amy-PET	Baseline plasma NfLs	All subjects: -↓ longitudinal hippocampal volume and global CTh -↓ longitudinal corpus callosum FA -↑ longitudinal FDG hypometabolism	Cross-sectional and longitudinal	Simoa (plasma), ELISA (CSF)
			Longitudinal plasma NfLs	All subjects: ↑ longitudinal amyloid burden		
Moore et al. (2018) [81]	70 MCI, 77 HC	WM	CSF NfLs	All subjects: ↓ cross-sectional striatum FA; ↑ cross-sectional striatum RD, fusiform gyrus MD, and rectus gyrus AxD MCI: ↓ cross-sectional superior corona radiata and posterior thalamic radiation FA, ↑ cross-sectional anterior corona radiata MD and AxD and striatum RD HC: ↑ cross-sectional posterior thalamic radiation RD	Cross-sectional	ELISA (CSF)
Moore et al. (2020) [82]	71 MCI, 82 HC	CTh, GM	CSF NfLs	All subjects: ↓ cross-sectional parieto-temporal CTh and fronto-temporo-cingulate GM volume MCI: ↓ cross-sectional temporo-parietal CTh and fronto-temporo-cingulate GM volume	Cross-sectional and longitudinal	ELISA (CSF)
Nabizadeh et al. (2022) [83]	92 MCI from ADNI database (47 ApoE ε4+, 45 ApoE ε4-)	WM	Plasma NfLs	ApoE ε4+: ↓ cross-sectional internal capsule, IFOF, fornix, and corpus callosum FA; ↑ cross-sectional corona radiata and sagittal stratum AxD, corona radiata, internal capsule, corpus callosum, IFOF, and fornix RD, and corona radiata, internal capsule, corpus callosum, and fornix MD ApoE ε4-: ↓ cross-sectional corona radiata FA; ↑ cross-sectional hippocampal cingulum, internal capsule, and UF AxD, and cingulum, hippocampal cingulum and UF RD and MD	Cross-sectional	Simoa (plasma)
Osborn et al. (2018) [84]	71 MCI, 77 HC	WMHs	CSF NfLs	All subjects: ↑ cross-sectional WMH volume	Cross-sectional	ELISA (CSF)
Shi et al. (2019) [85]	68 amnesic MCI, 87 HC	GM	Plasma NfLs	Amnesic MCI: ↓ cross-sectional temporal GM volume	Cross-sectional	Simoa (plasma)
Sun et al. (2020) [86]	675 MCI, 354 HC from ADNI database (589 longitudinal MRI)	WMHs	Baseline plasma NfLs	All subjects: ↑ cross-sectional and longitudinal WMH volume HC: ↑ longitudinal WMH volume	Cross-sectional and longitudinal	Simoa (plasma)
			Longitudinal plasma NfLs	All subjects: ↑ longitudinal WMH volume HC: ↑ longitudinal WMH volume		
Xie et al. (2023) [87]	361 MCI, 245 HC from ADNI database	GM	Plasma NfLs	All subjects: ↓ longitudinal perirhinal cortex GM volume	Cross-sectional and longitudinal	Simoa (plasma)

AD: Alzheimer’s disease; ADNI: Alzheimer’s disease neuroimaging initiative; AIBL: Australian imaging biomarkers and lifestyle study of ageing; ALS: amyotrophic lateral sclerosis; amy-PET: amyloid-positron emission tomography; ApoE: apolipoprotein E; APP: amyloid precursor protein gene; AxD: axial diffusivity; Aβ: β-amyloid; bvFTD: behavioral variant frontotemporal dementia; CA4-DG: cornu ammonis 4-dentate gyrus; CBS: corticobasal syndrome; CSF: cerebrospinal fluid; CTh: cortical thickness; C9ORF72: chromosome 9 open reading frame 72; ECLIA: electrochemiluminescence immunoassay; ELISA: enzyme-linked immunosorbent assay; ELLA: enzyme-linked lectin assay; FA: fractional anisotropy; FDG-PET: fluorodeoxyglucose-positron emission tomography; FTD: frontotemporal dementia; GM: grey matter; GRN: progranulin gene; HC: healthy controls; IFOF: inferior fronto-occipital fasciculus; ILF: inferior longitudinal fasciculus; lvPPA: logopenic variant primary progressive aphasia; MAPT: microtubule-associated protein tau gene; MCI: mild cognitive impairment; MD: mean diffusivity; MRI: magnetic resonance imaging; MSA: multiple system atrophy; MTA score: medial temporal lobe atrophy score; NODDI: neurite orientation dispersion and density imaging; NfLs: neurofilaments light chain; nfvPPA: non-fluent variant primary progressive aphasia; PA: posterior atrophy score; PD: Parkinson’s disease; PPA: primary progressive aphasia; PSEN1/2: presenilin 1/2 gene; PSP: progressive supranuclear palsy; RD: radial diffusivity; ROI: region of interest; SCD: subjective cognitive decline; Simoa: single molecule array; SLF: superior longitudinal fasciculus; svPPA: semantic variant primary progressive aphasia; tau-PET: tau-positron emission tomography; TRIAD: translational research informatics and data-management grid; UF: uncinate fasciculus; VaD: vascular dementia; VBM: voxel-based morphometry; WM: white matter; WMHs: white matter hyperintensities. ^1^ Familial AD: APP, PSEN1, PSEN2 mutations carriers. ^2^ Genetic FTD: C9ORF72 repeat expansion, GRN and MAPT mutations carriers.

## Data Availability

This is a scoping review of previously published records and all records are in the public domain.
